# Multiple Duties for Spindle Assembly Checkpoint Kinases in Meiosis

**DOI:** 10.3389/fcell.2017.00109

**Published:** 2017-12-13

**Authors:** Adele L. Marston, Katja Wassmann

**Affiliations:** ^1^Wellcome Centre for Cell Biology, Institute for Cell Biology, University of Edinburgh, Edinburgh, United Kingdom; ^2^Sorbonne Universités, UPMC Univ Paris 06, Institut de Biologie Paris Seine, UMR7622, Paris, France; ^3^Centre National de la Recherche Scientifique, Institut de Biologie Paris Seine, UMR7622 Developmental Biology Lab, Paris, France

**Keywords:** meiosis, spindle assembly checkpoint, Mps1, Bub1, BubR1/Mad3, Aurora B-like kinases, cohesin protection, chromosome congression

## Abstract

Cell division in mitosis and meiosis is governed by evolutionary highly conserved protein kinases and phosphatases, controlling the timely execution of key events such as nuclear envelope breakdown, spindle assembly, chromosome attachment to the spindle and chromosome segregation, and cell cycle exit. In mitosis, the spindle assembly checkpoint (SAC) controls the proper attachment to and alignment of chromosomes on the spindle. The SAC detects errors and induces a cell cycle arrest in metaphase, preventing chromatid separation. Once all chromosomes are properly attached, the SAC-dependent arrest is relieved and chromatids separate evenly into daughter cells. The signaling cascade leading to checkpoint arrest depends on several protein kinases that are conserved from yeast to man. In meiosis, haploid cells containing new genetic combinations are generated from a diploid cell through two specialized cell divisions. Though apparently less robust, SAC control also exists in meiosis. Recently, it has emerged that SAC kinases have additional roles in executing accurate chromosome segregation during the meiotic divisions. Here, we summarize the main differences between mitotic and meiotic cell divisions, and explain why meiotic divisions pose special challenges for correct chromosome segregation. The less-known meiotic roles of the SAC kinases are described, with a focus on two model systems: yeast and mouse oocytes. The meiotic roles of the canonical checkpoint kinases Bub1, Mps1, the pseudokinase BubR1 (Mad3), and Aurora B and C (Ipl1) will be discussed. Insights into the molecular signaling pathways that bring about the special chromosome segregation pattern during meiosis will help us understand why human oocytes are so frequently aneuploid.

## General introduction into meiosis

All sexually reproducing organisms rely on a specialized form of cell division known as meiosis, the defining feature of which is the generation of gametes with half the number of chromosomes of the parental cell. In contrast to the mitotic divisions, where ploidy is maintained by alternate rounds of DNA replication and chromosome segregation; during meiosis, replicated chromosomes are segregated twice successively, reducing ploidy by half. In most organisms, including humans and the model organisms discussed in this review, maternal and paternal chromosomes (homologs) are segregated during meiosis I, while the copies generated during DNA replication (sister chromatids) are segregated only during meiosis II, reminiscent of mitosis (reviewed in Marston and Amon, 2004). Perhaps as a result of the added complexity of successively segregating chromosomes, meiosis is highly error-prone. In human females, up to ~30% of all female gametes (oocytes/eggs) carry the incorrect number of chromosomes (Hassold and Hunt, 2001). Although such aneuploidy is a major cause of miscarriages, infertility and birth defects, the underlying molecular lesions are not well-understood. In part, this is due to a lack of knowledge about the pathways that orchestrate the meiotic divisions. Indeed, compared to mitosis, where intensive research over many years has shaped our understanding, our mechanistic knowledge of meiosis is less developed. In recent years, however, it has become apparent that many regulators of the mitotic cell cycle take on increased significance during meiosis. This is to some extent because meiosis is especially reliant on the canonical processes they control, but also because mitotic regulators gain novel functions during meiosis, in part through establishment of distinct interactions. This is particularly true for cell cycle-relevant kinases, which adopt a wide range of novel and canonical functions to control meiosis. Here, we focus on kinases of the central surveillance pathway in mitotic cells: the spindle assembly checkpoint (SAC) and review their roles in meiosis.

### Model systems to study meiotic divisions

Our knowledge of meiosis is derived from a rich array of model organisms. Pathways of homolog recognition and meiotic recombination have particularly benefited from a long history of complementary studies in diverse organisms. We focus here on studies of the SAC kinases in the yeasts *Saccharomyces cerevisiae* and *Schizosaccharomyces pombe*, and mouse oocytes. For clarity, gene names relating to different organisms are given in the order: *S. cerevisiae*/*S. pombe*/mouse.

#### Budding yeast and fission yeast

**Figure 1:**
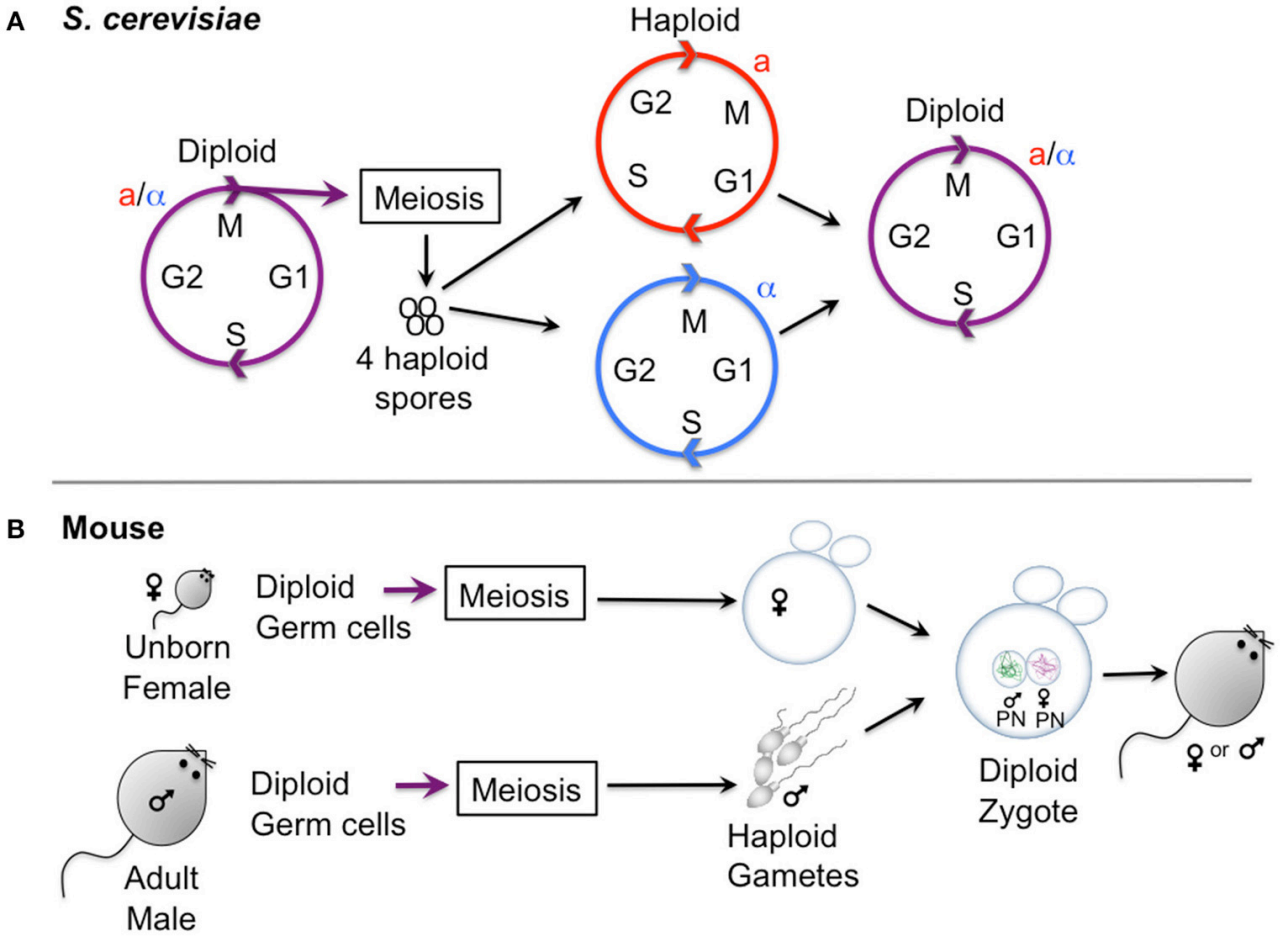
Gametogenesis in yeast and female mice. **(A)** Outline of the lifecycle of the budding yeast *S. cerevisiae*, which can propagate vegetatively through mitotic division in both its haploid and diploid form. Haploid yeast of opposite mating types (a and alpha) mate and undergo conjugation to generate a zygote, upon which nuclei fuse (karyogamy). Starvation of diploid cells triggers meiosis, culminating in the production of four haploid spores. Note that the lifestyle of *S. pombe* is similar, except that meiosis and sporulation occur directly after karyogamy so that diploid cells are short-lived. **(B)** Oocyte differentiation in female mice. Gametes are generated from diploid germ cells, in the female embryo before birth for oocytes, and in the adult male for spermatocytes. After hormonal stimulation in adult females, some oocytes that are arrested in prophase I undergo a growth phase. During the menstrual cycle usually one (humans) or several (mouse) oocytes are induced to undergo meiosis I and enter meiosis II, where they will remain arrested to await fertilization with a male gamete that has already finished meiosis I and II and has progressed into G1 phase. Oocytes exit meiosis II after fertilization, and the male and female pronucleus fuse to form the diploid zygote, the first cell of the embryo.

Unicellular fungi, in particular the budding yeast, *Saccharomyces cerevisiae*, and the fission yeast, *Schizosaccharomyces pombe*, have provided a wealth of basic information relating to chromosome segregation during meiosis. *S. cerevisiae* undergoes a vegetative (mitotic) cycle as a haploid, existing in two mating types (sexes), known as a and alpha, which can mate to form an a/alpha diploid, which can also undergo a vegetative cycle (Figure 1). Upon starvation, diploids will undergo meiosis to generate four haploid spores. *S. pombe* similarly exists in two haploid sexes (h+ and h−) that undergo conjugation and meiosis to generate four haploid spores. The ability to isolate both haploids and diploids of yeasts enables ease of genetic manipulation. One advantage of both yeasts for studying meiosis is that all four products of meiosis can be isolated, so that chromosome segregation can be recapitulated. Furthermore, a wide range of genetic tools exist. This includes the ability to label individual chromosomes with fluorescent markers (Straight et al., 1996; Michaelis et al., 1997) and methods to highly synchronize cell populations (Bähler et al., 1991; Carlile and Amon, 2008; Kakui et al., 2011; Cipak et al., 2012; Chia and van Werven, 2016). Yeast also have the advantage that large quantities can easily be grown for biochemical analyses, yet they are equally amenable to single cell live cell imaging.

#### Mouse oocytes

Most of our knowledge on the regulation of meiotic divisions in mammals has been obtained from mouse oocytes. Male division in meiosis cannot currently be dynamically followed in the same manner as female meiosis, therefore we focus here on female meiosis. Mammalian oocytes are generated in the female embryo from germinal cells that form the primordial follicle. Until sexual maturity, oocytes are maintained arrested in prophase I until—depending on the species—a variable number are hormonally stimulated to grow and enter the first meiotic division (El Yakoubi and Wassmann, 2017). To study murine oocyte cell division in meiosis I and II, mature prophase I arrested oocytes that have finished their growth phase can be obtained from adult, female mice. Oocytes are usually harvested in medium that maintains the prophase arrest by inhibiting the signaling pathway inducing a rise in Cdk1 associated kinase activity, such as for example medium containing dbcAMP, which maintains the kinase PKA active, preventing Cdk1 activation. This is necessary, because mammalian oocytes (unlike *Xenopus laevis* oocytes for example) spontaneously enter the first meiotic division upon removal of the follicle cells surrounding them. Mouse oocytes that have been cleaned from follicle cells are then released into medium allowing maturation. Under normal culture conditions, oocytes undergo Germinal Vesicle breakdown (GVBD), which is visible under the microscope, in a very synchronized manner, 45–90 min after release. The metaphase-to-anaphase transition of the first meiotic division takes place around 7–10 h after GVBD, depending on the mouse strain that is being used. Exit from meiosis I is visible due to the extrusion of a small Polar Body (PB). Oocytes then progress into meiosis II, where they remain arrested in metaphase II to await fertilization. This arrest point is called CSF, or Cytostatic Factor arrest (Perry and Verlhac, 2008). It is only upon fertilization that oocytes finalize the second meiotic division *in vivo*, but *in vitro* cultured oocytes in metaphase II can be artificially “activated” to undergo the metaphase II-to-anaphase II transition. The high synchrony and temporal resolution of the meiotic divisions permits harvesting pools of oocytes at all meiotic cell division stages for fixation or preparation of cell extracts, or their observation by live imaging.

Mouse oocytes are transparent and can be injected with fluorescent proteins to follow, for example, chromosome movements or the localization of kinetochore proteins. Conditional knock-out approaches using oocyte-specific promoters such as *Zona pellucida 3* (Zp3) to express *Cre* recombinase, are successful to knock-out genes during oocyte growth before entry into meiosis I (Lewandoski et al., 1997; Lan et al., 2004). This approach can be used for gene products that are otherwise essential for cell division, and that are not stable throughout the different meiotic stages before entry into meiosis I. Oocytes can be fixed for immunostaining or chromosome spreads, but they are not suitable for more sophisticated biochemical assays, due to the small oocyte count per mouse and the low amount of protein per oocyte. Alternative model systems have to be used for purification of proteins or activity assays.

### Mitotic and meiotic divisions: specialization of the chromosome segregation machinery during meiosis

The generation of gametes with half the chromosome content to somatic cells requires the remodeling of the chromosome segregation machinery. It is useful to consider mitotic chromosome segregation before discussing the modifications that are superimposed on the canonical machinery for execution of the meiotic divisions.

#### Mitosis

Following DNA replication during mitosis, the two newly duplicated sister chromatids are held tightly together by a ring-shaped protein complex, called cohesin, which is postulated to provide this cohesion by entrapping the sister chromatids topologically within (reviewed in Nasmyth, 2011). In mammals, but not yeast, the bulk of cohesin is removed from chromosome arms due to the action of Wapl, acting in the so-called prophase pathway. However, a fraction of cohesin is retained, primarily at the centromeres to ensure that sister chromatids remain associated. Pericentromeric shugoshin (Sgo1) protein, counteracts Wapl1 activity to ensure retention of cohesin in this region. At metaphase, the sister kinetochores assembled on the centromeres attach to spindle microtubules that emanate from opposite poles of the cell and cohesin counteracts the resultant pulling forces. The SAC and error correction machinery (see below) together monitor this attachment, delaying anaphase onset until all chromosomes have achieved the stable biorientation of sister chromatids. Once this has occurred, the SAC is satisfied and the enzyme, separase, becomes active and cleaves all remaining cohesin along the entire length of chromosomes, destroying the links between sister chromatids and initiating their poleward movement.

#### Meiosis

**Figure 2:**
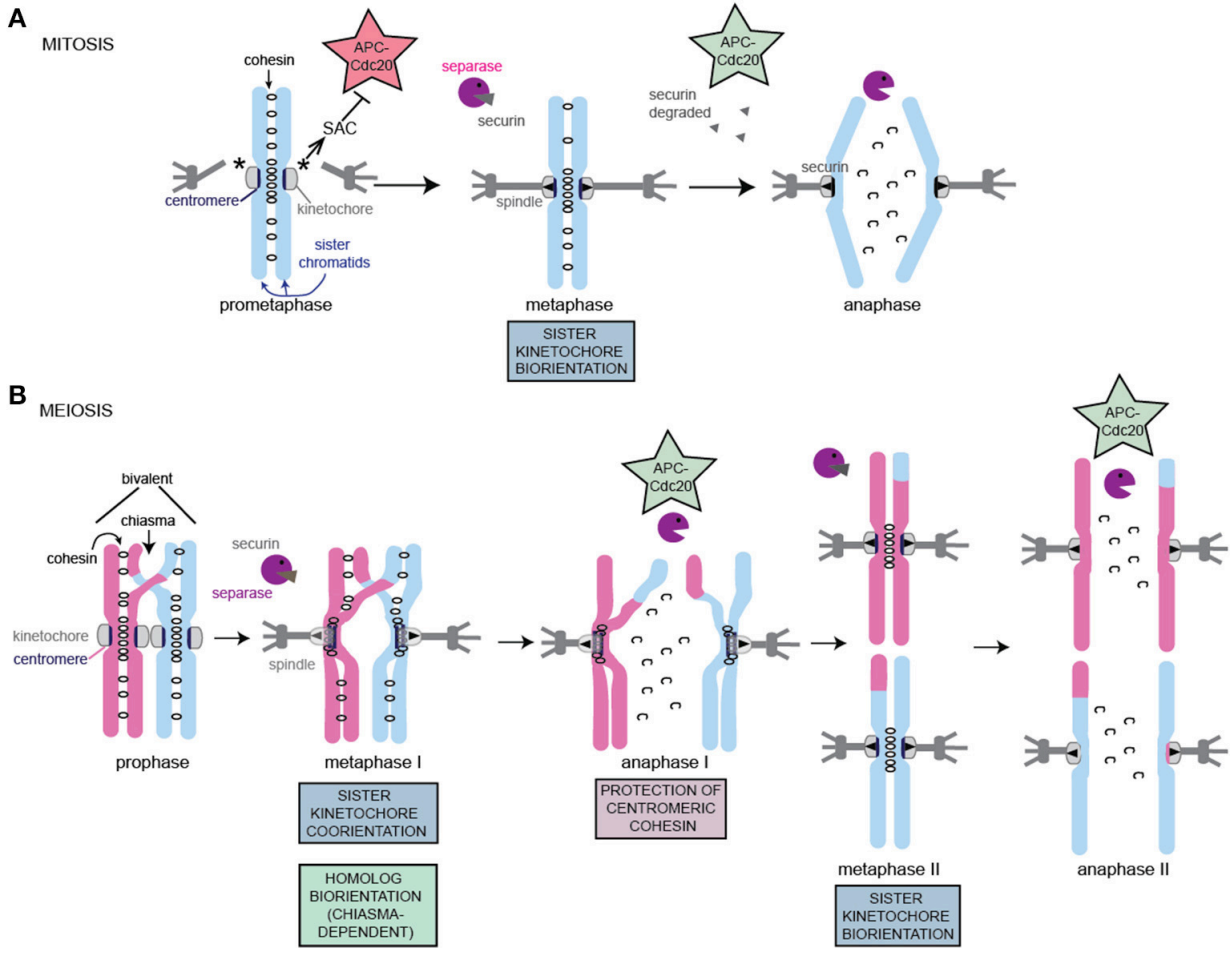
Schematic of chromosome segregation during mitosis and meiosis. The key adaptations to meiotic chromosomes are indicated. Note that while a single microtubule contacts each kinetochore (mitosis, meiosis II) or pair of kinetochores (meiosis I) in *S. cerevisiae* in most organisms, multiple microtubules connect to each kinetochore, resulting in increased probability and configurations of incorrect attachments in both mitosis and meiosis. **(A)** Key features of mitotic chromosome segregation. **(B)** Key features and adaptations of meiotic chromosome segregation. For details see text.

The segregation of homologs during meiosis I, followed by sister chromatids during meiosis II requires that the canonical chromosome segregation machinery is adapted. As in mitosis, chromosomes are replicated and cohesin is established between them. Subsequently, two consecutive chromosome segregation events occur. In addition to poorly understood changes to cell cycle controls, which not only ensure that meiosis I is followed not by interphase, but by meiosis II, the configuration of chromosomes is different in 3 major ways (Figure 2) (Marston and Amon, 2004). First, homologous chromosomes must be linked together to enable them to attach to microtubules in a tension-generating configuration. This requires homologs to pair with their partner and, in most organisms, they will undergo crossover recombination to produce chiasmata and generate a bivalent (consisting of 4 chromatids, Figure 2) (reviewed in Hunter, 2015). Chiasmata, together with cohesion between sister chromatids on chromosome arms distal to the chiasmata, hold the homologs together in this bivalent. This is because, once DNA repair is complete, a chiasma constitutes two DNA strands, which cross, but are not physically associated themselves. Rather, the recombinant sister chromatid (blue and pink in Figure 2) makes cohesive contacts not only with its identical sister chromatid but, also distal to the chiasmata, with its homologous chromatid, thereby linking the homologs and stabilizing the bivalent. Second, in contrast to mitosis, or meiosis II, where sister kinetochores attach to microtubules from opposite poles (sister kinetochore bi-orientation); during meiosis II, sister kinetochores attach to microtubules from the same pole (co-orientation, also called mono-orientation). In budding yeast, maize and flies, fusion of sister kinetochores appears to underlie their co-orientation (Goldstein, 1981; Li and Dawe, 2009; Sarangapani et al., 2014), however recent data suggest that this may not be the case in human oocytes (Patel et al., 2015; Zielinska et al., 2015). Third, cohesin, the ring shaped complex which is established during DNA replication to hold sister chromatids together, is lost in two steps during meiosis. During meiosis I, cohesin is proteolytically cleaved along chromosome arms by the enzyme separase. Because sister chromatid cohesion on chromosome arms distal to chiasmata is the only thing holding the homologs together, this triggers their segregation to opposite poles. However, cohesin is maintained in the region surrounding the centromeres, which ensures that sister chromatids remain linked until meiosis II. Protection of pericentromeric cohesin from separase activity requires a complex of shugoshin and protein phosphatase 2A, which are localized in the pericentromere and here act to prevent the proteolytic loss of cohesin, rather than Wapl-dependent removal as in mammalian mitosis (reviewed in Marston, 2015; specific aspects discussed below).

It is also important to note here that mammalian oocytes, and those of other animal species such as *Drosophila*, also encounter additional challenges to segregate chromosomes compared to yeasts in building a bipolar spindle and attaching kinetochores to microtubules. This is because oocytes are acentrosomal, and instead the chromosomes themselves organize the establishment of a bipolar spindle (Ohkura, 2015). Furthermore, the number of microtubule binding sites on each kinetochore is greater in mammalian oocytes, increasing the complexity of kinetochore-microtubule attachments (*S. cerevisiae*, in contrast, have only a single microtubule-binding site per kinetochore; Winey et al., 1995).

### Consequences of mis-segregation in meiosis

Two haploid gametes fuse to give rise to a diploid cell -the zygote- which is the first cell of the future embryo. If one of the gametes does not harbor the correct chromosome count, the resulting embryo will be aneuploid. Most aneuploidies are not viable, and in humans only certain trisomies, or monosomies of the sex chromosomes, are viable. Therefore, human aneuploid embryos are spontanously aborted in the first trimester and increased aneuploidy rates in meiosis affect pregnancy outcome. In humans, the high aneuploidy rate in female meiosis has important consequences for achieving a successful pregnancy. Mis-segregation rates in meiosis increase sharply with maternal age, with women closer to menopause having a risk of more than 30 % of being pregnant with a trisomic baby (Hassold and Hunt, 2001; El Yakoubi and Wassmann, 2017).

The reasons for the high error rate in meiotic chromosome segregation are multiple: Oocytes are not replenished with age, and before entry into the first meiotic division they may have been arrested in prophase for several decades. It has been shown in mice that there is no turn-over of cohesins during the extended prophase I arrest where oocytes await hormonal stimulation to grow and divide (Revenkova et al., 2010; Tachibana-Konwalski et al., 2010). Cohesins deteriorate with age and this leads to less solid connections between sister chromatids and as a consequence, weakening and even loss of chiasmata (Chiang et al., 2010; Lister et al., 2010). Once oocytes enter meiosis I, sister chromatids which are not held together by chiasmata cannot attach correctly and will biorient (Jessberger, 2012). The SAC may not be able to distinguish monopolar attachments of sister kinetochores of a bivalent from the bipolar attachment of a single chromosome, as both generate tension that will stabilize end-on attachments. Additionally, it was shown in human oocytes that recruitment of the SAC factors Bub1 and BubR1 to kinetochores decrease with age indicating that the SAC is less able to recognize missing attachments and to induce a cell cycle arrest (Lagirand-Cantaloube et al., 2017). This decrease in addition to the inherent leakiness of the meiotic checkpoint, which requires several unattached/tensionless kinetochores to mount a robust SAC dependent delay, could additionally lead to the observed increased error rate in oocytes of aged mothers.

Loss of cohesin with age not only leads to loss of chiasmata resulting in biorientation of unpaired chromosomes in meiosis I, but can also allow the precocious separation of sister chromatids. In mice it was shown that recruitment of the centromeric cohesin protector, Sgo2, to the centromere region also diminishes with age, and this has been correlated with premature loss of cohesin and therefore separation of some sister chromatids in meiosis I instead of in meiosis II (Lister et al., 2010). Such uncohered sister chromatids cannot be separated correctly in meiosis II, however, a rescue mechanism, whereby sister chromatids that have undergone recombination are preferentially segregated into the oocytes, rather than the polar body, has recently been discovered in humans (Ottolini et al., 2015). This could represent a form of “meiotic drive,” a phenomenon whereby certain traits are preferentially transmitted to the offspring.

### Spindle assembly checkpoint control

The specialised chromosome segregation pattern in meiosis also requires the adaptation of control mechanisms that regulate cell divisions in somatic cells. One control mechanism of significant importance during meiotic cell division in mammalian oocytes is the spindle assembly checkpoint or SAC, which ensures that chromosome segregation takes place only after correct end-on kinetochore microtubule attachments to both poles have been achieved (Wassmann et al., 2003; Homer et al., 2005; Niault et al., 2007; McGuinness et al., 2009; Hached et al., 2011). Molecular details on checkpoint response have been mainly discovered in mitotic cells of different model organisms, thus mitotic SAC control will be shortly introduced below before discussing the specificities of this checkpoint in meiosis.

In mitosis, paired sister chromatids have to be faithfully segregated into two daughter cells. Failures in the correct partitioning of the genetic material will lead to the generation of aneuploid daughter cells which harbor an incorrect number of chromosomes. Therefore it is pivotal that separase is not activated to remove cohesin before all sister chromatids are correctly attached with their kinetochores to the bipolar spindle. It is the job of the SAC to ensure that anaphase onset takes place only when stable end-on attachments that are under tension are present at each kinetochore. Tension applied by the opposite poles of the spindle further stabilizes attachments. In short, the SAC recognizes the presence of kinetochores that harbour unoccupied microtubule binding sites, and if this is the case, delays the metaphase-to-anaphase transition by inhibiting the anaphase promoting complex/cyclosome (APC/C) coupled to its activator, Cdc20. APC/C^Cdc20^ activation will result in the degradation of the separase inhibitor, securin, resulting in cohesin cleavage and is essential for chromatid segregation in mitosis, and chromosome segregation in meiosis I in *S. cerevisiae, Xenopus* and mouse oocytes (Herbert et al., 2003; Terret et al., 2003; Lee et al., 2004; Kudo et al., 2006; Zhang et al., 2008; Note that this was initially controversial in *Xenopus*, see discussion in Zhang et al., 2008). APC/C^Cdc20^ inhibition by the SAC depends on the phosphorylation of the SAC components Bub1, Mad1, the kinetochore protein Knl1 by the kinase Mps1, and binding of Mad2 to attachment sites on the kinetochore that are devoid of stable microtubule binding. Mps1 localizes on and off the microtubule binding sites on the unattached kinetochores as long as there are no stably bound microtubules, thereby maintaining SAC activity. Continuous cycling of cytosolic Mad2 between active and inactive conformations by binding to Mad1/Mad2 complexes that are more stably associated with unattached kinetochores leads to the generation of the diffusible MCC (mitotic checkpoint complexes), which binds to APC/C^Cdc20^ to induce metaphase arrest (for more details see excellent reviews (Khodjakov and Pines, 2010; Musacchio, 2015; Sacristan and Kops, 2015). In *S. cerevisiae* and *Drosophila*, SAC control is essential only upon conditions when proper microtubule-kinetochore attachments are perturbed, for example after treatment with microtubule depolymerizing drugs or the introduction of mutants affecting establishment of a proper spindle (Hoyt et al., 1991; Li and Murray, 1991; Buffin et al., 2007). In mammals, the checkpoint is active during each cell cycle as cells enter mitosis and progress through prometaphase, and prevents accelerated progression through early steps of mitosis (Taylor and McKeon, 1997; Gorbsky et al., 1998; Dobles et al., 2000; Kalitsis et al., 2000). SAC control is therefore essential to generate euploid daughter cells under unchallenged conditions. The SAC is gradually shut off as cells progress into metaphase and end-on attachments are established. In the absence of a functional SAC, progression through mitosis is accelerated (Meraldi et al., 2004; Tighe et al., 2008; Sliedrecht et al., 2010). As long as anaphase onset did not occur, the checkpoint can be re-activated, and cells re-establish a metaphase arrest (Collin et al., 2013; Kamenz and Hauf, 2014; Vázquez-Novelle et al., 2014).

Once separase becomes active and removes cohesin at anaphase onset, attachments are no longer under tension. SAC activity depends on elevated Cyclin B-Cdk1 levels, therefore once anaphase onset occurs and Cyclin B1 becomes degraded, the SAC can no longer be re-activated. Hence, the metaphase-to-anaphase transition is regulated by a bistable switch through two feedback loops, ensuring the irreversibility of anaphase onset (He et al., 2011).

On a molecular level it is known that the cell cycle arrest in metaphase upon SAC activation is brought about by the generation of the MCC, which consists of the core SAC components Mad2, BubR1 and Bub3, and the APC/C activator Cdc20 (Fraschini et al., 2001; Sudakin et al., 2001; Tang et al., 2001). The MCC prevents ubiquitination and hence degradation of two key APC/C substrates at the metaphase-to-anaphase transition, namely Cyclin B1 and securin. In vertebrates, both proteins keep separase inactive, and their stabilization upon checkpoint activation thus prevents cohesin removal in metaphase, and anaphase onset. Several kinases and one pseudokinase are involved in SAC control (Sacristan and Kops, 2015), with the dual specificity kinase Mps1 playing a pivotal role, which will be outlined in detail below. The kinase Bub1 is essential for SAC functioning, but independently of its kinase activity. Mammalian BubR1 is a pseudo-kinase, homologous to yeast Mad3 and an essential component of the MCC.

Working in conjunction with the SAC is the “error correction” system, which detects tension-less kinetochore-microtubule attachments and severs them, thereby providing a further opportunity for correct, tension-generating attachments to be established. Aurora B kinase activity is central to this process, working by directly phosphorylating components at the interface of the microtubule-kinetochore interaction to abolish it (see Krenn and Musacchio, 2015 for a review on the various kinetochore substrates). This in turn, creates unattached kinetochores, which activate the SAC. Whether Aurora B is also part of the SAC signaling cascade *per se* is still controversial (Santaguida et al., 2011; Gurden et al., 2016). Because Aurora B participates in proper Mps1 localization, it is at least indirectly required for SAC signaling (Maldonado and Kapoor, 2011; Saurin et al., 2011).

### SAC control in meiosis

The meiotic divisions in oocytes are highly error prone and have led to speculations on the inefficiency of SAC control in meiosis. In *S. cerevisiae*, loss of Mad2 leads to an acceleration of meiosis I with premature APC/C activation, and the generation of aneuploid spores, due in part to the mis-segregation of homologs during meiosis I (Shonn et al., 2000, 2003; Cheslock et al., 2005; Lacefield and Murray, 2007; Tsuchiya et al., 2011). Given that *MAD2* is not essential for viability in *S. cerevisiae*, this indicates a much greater requirement for the SAC in the accurate segregation of homologs during meiosis I than in mitosis. In contrast, *Xenopus laevis* oocytes have been reported to divide without being able to mount a detectable checkpoint delay, and despite this the error rate of chromosome segregation is not worse than in mammalian oocytes, which can induce a SAC delay (Shao et al., 2013; Liu et al., 2014). In *Drosophila* oocytes, the SAC components Mps1 and BubR1 are required for correct chromosome segregation in meiosis I (Gilliland et al., 2005; Malmanche et al., 2007), but according to one study, without detectable influence on APC/C activity (Batiha and Swan, 2012), and therefore potentially independently of the SAC. However, SAC proteins are present in both *Drosophila* and *X. laevis* oocytes so it remains possible that they provide some function during meiosis in these organisms, too.

On the contrary, it is well established that mammalian oocytes possess a functional checkpoint leading to a detectable delay in anaphase I onset, when activated, even though the meiotic cell divisions are highly error prone (Wassmann et al., 2003; Homer et al., 2005; Niault et al., 2007; Hassold and Hunt, 2009; McGuinness et al., 2009; Hached et al., 2011). Mice harboring a conditional knock-out of any of the essential SAC components are sterile, because meiosis I is significantly accelerated and chromosome missegregations occur at elevated rates. The checkpoint kinases Mps1, Bub1, and the pseudokinase BubR1 are, as in mitosis, essential for SAC functioning (McGuinness et al., 2009; Hached et al., 2011; Touati et al., 2015). The meiotic checkpoint in oocytes is rather leaky (Gui and Homer, 2012; Lane et al., 2012; Sebestova et al., 2012), which may be related to the huge size of the oocyte on the one hand, and the specificities of the meiotic divisions as far as kinetochore orientation is concerned, on the other hand. The molecular details of SAC control in meiosis compared to mitosis are still not entirely clear, as well as the question of what kind of errors are detected in meiosis compared to mitosis. Because we want to focus here on the less-known roles of checkpoint kinases in meiosis we refer the reader to more specialized reviews on the meiotic SAC for more detailed information (Jones and Lane, 2013; Gorbsky, 2015; Touati and Wassmann, 2016). But the fact that SAC proteins are present in oocytes of organisms that seem not to be able to mount a detectable checkpoint delay in anaphase I onset is maybe a hint that additional, essential roles in meiosis have put evolutionary pressure on maintaining SAC proteins expressed in oocytes.

## Kinases in detail

### Distinct requirements for Bub1 kinase activity to protect centromeric cohesin in yeast and mammals ?

The S/T kinase Bub1 is an essential conserved SAC component in mitosis and meiosis. Interestingly, the kinase activity of Bub1 is not required for checkpoint arrest in mouse oocytes (McGuinness et al., 2009; Vleugel et al., 2015; El Yakoubi et al., 2017), as in both *S. cerevisiae* and *S. pombe* mitosis (Vaur et al., 2005; Fernius and Hardwick, 2007). Like other SAC proteins, Bub1 protein localizes dynamically to unattached kinetochores through interaction with Bub3. Its main role in the SAC is the recruitment of Bub3-BubR1 and Cdc20 to unattached kinetochores. Contrary to the closely related BubR1 pseudo-kinase, Bub1 does not get incorporated into the MCC. Its role in the SAC is limited to promoting Bub3 binding to the kinetochore scaffold Spc105/Spc7/Knl1 that is phosphorylated by Mps1 in the absence of attachments. Bub1's role is thus essential for the recognition of the signal generated upon missing attachments (reviewed in Musacchio, 2015; Sacristan and Kops, 2015).

#### Bub1 in yeast meiosis

The first indication that Bub1 may play a role in meiosis that is distinct from its checkpoint function came from a study in *S. pombe* (Bernard et al., 2001). In contrast to *mad2*Δ cells, *bub1*Δ cells were found to prematurely separate sister chromatids and the meiotic cohesin subunit, Rec8, was removed from centromeres concomitant with arm cohesin during meiosis I (Bernard et al., 2001). This indicated that Bub1 is important for the protection of cohesion at centromeres in this organism. At least in *S. pombe*, the kinase activity of Bub1 was found to be important for localization of Sgo1 during meiosis (Kawashima et al., 2010). An explanation for these findings came with the discovery of shugoshins in both *S. pombe* and *S. cerevisiae* (Katis et al., 2004; Kitajima et al., 2004; Marston et al., 2004; Rabitsch et al., 2004). Shugoshins, along with protein phosphatase 2A, shield pericentromeric cohesin from separase during the first meiotic division (Brar et al., 2006; Kitajima et al., 2006; Riedel et al., 2006; Ishiguro et al., 2010; Katis et al., 2010). To achieve this specific protection, shugoshins must be localized to the pericentromeric region, and this is the key non-SAC role of Bub1 in both *S. pombe* and *S. cerevisiae* meiosis (Kitajima et al., 2004; Kiburz et al., 2005; Riedel et al., 2006). Bub1, itself localized on the kinetochore, phosphorylates histone H2A at a specific residue (S121 in yeasts) to create a binding site on the nucleosome for shugoshin (Sgo1). Later work has established an important role for this modification, and shugoshin also in mitotic cells (reviewed in Marston, 2015). During meiosis II in *S. cerevisiae*, however, Bub1 does not need to be retained at kinetochores for persistence of pericentromeric shugoshin-PP2A (Argüello-Miranda et al., 2017). Instead evidence suggests that Mps1 is the critical kinase in localizing shugoshin-PP2A during meiosis II in budding yeast Argüello-Miranda et al., 2017; see below).

#### Bub1 in oocytes

Complete loss of Bub1 in oocytes leads to gross missegregations, strong acceleration of prometaphase I and sterility (McGuinness et al., 2009). Importantly, SAC control can be rescued by expressing a kinase-dead version of Bub1 in oocytes, showing that also in oocytes, the kinase activity of Bub1 is not required for SAC signaling (McGuinness et al., 2009).

In addition to its role in the SAC, Bub1 kinase activity was shown to be required for chromosome congression in somatic cells (Ricke et al., 2012; Asghar et al., 2015). Rescue experiments of Bub1-conditional knock-out oocytes indicate that the kinase domain of Bub1 plays only a minor role for chromosome congression in oocytes (McGuinness et al., 2009). Loss of Bub1 kinase activity in oocytes conditionally expressing only a kinase-dead version of Bub1 results in an arrest in metaphase I, or anaphase I onset with a significant delay due to checkpoint activation, when these oocytes are cultured *in vitro* (El Yakoubi et al., 2017). *In vivo* matured oocytes devoid of Bub1 kinase activity eventually exit metaphase I arrest and progress into meiosis II where they can be fertilized. Bub1 kinase-dead mice are therefore fertile (Ricke et al., 2012). This result indicates that even though chromosome congression can in part be rescued by expressing Bub1 not harbouring any kinase activity, Bub1 protein still plays a role in stable attachment and alignment of chromosomes, and in its absence, the SAC is activated (El Yakoubi et al., 2017).

Besides its canonical role in the checkpoint and its role for chromosome congression, mammalian Bub1 generates a histone mark by phosphorylating T120 (human), or T121 (mouse) of H2A, in somatic and germ cells, similar to yeast. In mammalian mitosis this histone mark provides a direct binding site for Sgo1 at the kinetochore (Kawashima et al., 2010; Liu et al., 2015) and is required for focused localization of Sgo1 at the centromere, as in yeast (Tang et al., 2004; Kitajima et al., 2005; Kawashima et al., 2010; Liu et al., 2013b). Sgo1 is important during mitosis to protect centromeric cohesin from the non-proteolytic pathway of cohesin removal that occurs in mitotic prophase, at the hands of Wapl (Hauf et al., 2005; Kueng et al., 2006; Shintomi and Hirano, 2009; Liu et al., 2013b). However, in human mitotic cells, this protective function of Sgo1 is provided by a pool associated with cohesin in the inner centromere, rather than the Bub1 kinase-dependent H2A-bound kinetochore-associated pool. Bub1-dependent binding of Sgo1 to H2A does, however, play an indirect role in cohesin protection because it is a prerequisite for the transcription-dependent translocation to cohesin to the inner centromere (Liu et al., 2013a,b, 2015). By analogy with mitosis, it was assumed that the same histone mark would contribute to the protection of centromeric cohesin in meiosis, but this time to protect cohesin from cleavage by separase. Surprisingly though, the kinase activity of Bub1 and phosphorylation of H2A T121 are not required for Sgo2 localization and protection of centromeric cohesin in oocytes, even though Bub1 itself, independently of its kinase activity, contributes to meiotic Sgo2 localization and cohesin protection (McGuinness et al., 2009; El Yakoubi et al., 2017). Accordingly, mice harbouring only kinase-dead Bub1 are fertile, and oocytes do not missegregate sister chromatids. In contrast, complete loss of Bub1 in oocytes leads to some precocious sister chromatid segregation, in agreement with the fact that Bub1 protein independently of its kinase activity and phosphorylation of H2A participates in the centromeric recruitment of Sgo2 (McGuinness et al., 2009; El Yakoubi et al., 2017), Figure 3.

**Figure 3:**
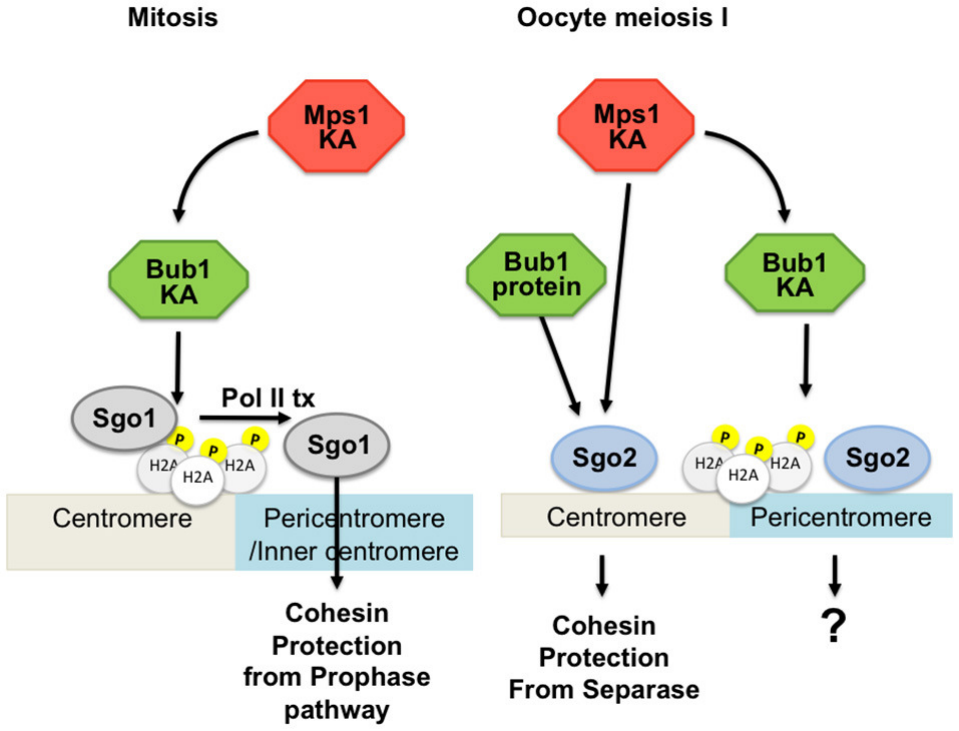
Involvement of Bub1 and Mps1 kinases in Sgo2 localization in mouse oocytes. On the left: Sgo1 in mitosis is localized by Bub1-dependent phosphorylation of H2A and transcription for protection of cohesin. On the right: Two pools of Sgo2 can be distinguished in oocyte meiosis I, one of which is localized independently of H2A phosphorylation by Bub1, and which is required for protection of centromeric cohesin. Mps1 kinase is involved in localizing the pool of Sgo2 required for protection, at the centromere. Bub1 kinase is required for localizing a pool of Sgo2 in an H2A-phosphorylation dependent manner, but this pool is largely dispensible for protection of centromeric cohesin in meiosis I. KA, kinase activity; Pol II tx, Polymerase II dependent transcription.

#### Summary

While the importance of Bub1 protein in both mitotic and meiotic divisions across species is clear, the requirement of its kinase activity differs. There is a general role for Bub1 kinases in phosphorylating Histone H2A to create a direct binding site for shugoshins. However, the importance of this Bub1 kinase-dependent histone H2A-bound shugoshin pool for cohesin protection in meiosis differs, being dispensible in mouse oocytes and required only during meiosis I, in *S. cerevisiae* (Table 1).

**Table 1:** Summary of the roles occupied by the different kinases in the SAC and outside the SAC, in distinct organisms (See text for details.).

**Kinase**	**Roles in SAC**	**Roles outside the SAC**		
		**Mitosis**	**Meiosis, *S. cerevisiae/S. pombe***	**Meiosis, Mouse oocytes**
Bub1	Promotes recruitment of Bub3-BubR1 and Cdc20 to unattached kinetochores. Kinase activity not required (Musacchio, 2015; Sacristan and Kops, 2015; Vleugel et al., 2015).	Phosphorylates Histone H2A on T120 for Sgo1 recruitment which contributes to protection of cohesin from prophase pathway (Tang et al., 2004; Kitajima et al., 2005; Kawashima et al., 2010; Liu et al., 2013b). Required for chromosomecongression (Ricke et al., 2012; Asghar et al., 2015).	Phosphorylates Histone H2A on S121 to create a mark for Sgo1 recruitment to the pericentromere, to protect cohesin from cleavage by separase, at least in meiosis I (Kitajima et al., 2004; Kiburz et al., 2005; Kawashima et al., 2010).	Phosphorylates H2A on T121 to localize Sgo2. This fraction of Sgo2 is not essential for protection from separase (El Yakoubi et al., 2017). Role for localizing Sgo2 for protection, independently of its kinase activity (McGuinness et al., 2009; El Yakoubi et al., 2017). Required for chromosome congression (McGuinness et al., 2009; El Yakoubi et al., 2017).
Mps1	Phosphorylates MELT repeats in the outer kinetochore scaffold Spc105/ Spc7/ Knl1, to promote MCC assembly (Musacchio, 2015; Sacristan and Kops, 2015). Required for Bub1 and BubR1 kinetochore recruitment (Musacchio, 2015; Sacristan and Kops, 2015).	Required for SPB duplication in *S. cerevisiae* (Winey et al., 1991). In mammalian cells required for full Aurora B activity and therefore error correction (van der Waal et al., 2012).	Required for SPB duplication (Straight et al., 2000), Conversion of lateral to end-on attachments (Meyer et al., 2013). Inactivation required for deprotection of centromeric cohesin in meiosis II (Jonak et al., 2017).	Required for correct localization of Aurora B (Hached et al., 2011), BubR1 (Touati et al., 2015), and Bub1 kinetochore localization (El Yakoubi et al., 2017). Kinase activity required for localization of Sgo2 and centromeric cohesin protection (El Yakoubi et al., 2017).
Mad3/BubR1	Pseudokinase (BubR1), or no kinase domain (Mad3) (Suijkerbuijk et al., 2012) Component of the MCC (Fraschini et al., 2001; Sudakin et al., 2001; Tang et al., 2001). Binds unattached kinetochores via Bub3 and MELT repeats phosphorylated on Knl1 by Mps1 (Musacchio, 2015; Sacristan and Kops, 2015).	BubR1 recruits PP2A to kinetochores, where it counteracts Aurora B substrate phosphorylation and thereby stabilizes microtubule- kinetochore interactions (Suijkerbuijk et al., 2012; Kruse et al., 2013; Xu et al., 2013).	Not essential for meiosis. Becomes essential when non-exchange chromosome present, and induces a prophase I delay (Shonn et al., 2000, 2003).	Promotes progression through anaphase I (Touati et al., 2015). Is not required for prophase I arrest (Touati et al., 2015).
Ipl1/Ark1/Aurora B/C	Aurora B contributes to correct Mps1 kinetochore localization (Maldonado and Kapoor, 2011; Saurin et al., 2011). Role in mammalian SAC control controversial (Santaguida et al., 2011; Gurden et al., 2016).	Aurora B and Ipl1 phosphorylate proteins at the kinetochore- microtubule interface and thereby destabilize them (Krenn and Musacchio, 2015). Aurora B promotes lateral attachments in early mitosis, controls lateral to end-on conversion (Kalantzaki et al., 2015; Shrestha et al., 2017). Required for chromosome condensation and cytokinesis (van der Horst and Lens, 2014).	Ipl1 is required for conversion of lateral to end-on attachments (Meyer et al., 2013), and error correction (Monje-Casas et al., 2007; Meyer et al., 2013), it prevents spindle assembly in prophase I (Shirk et al., 2011; Kim et al., 2013; Newnham et al., 2013), and promotes kinetochore remodeling in prophase I (Kim et al., 2013; Meyer et al., 2015). Ipl1 has furthermore a role in maintaining centromeric cohesion (Monje-Casas et al., 2007; Yu and Koshland, 2007), Ark1 promotes sister kinetochore co-orientation (Hauf et al., 2007; Sakuno et al., 2011).	Aurora C mainly responsible for localized CPC activity, chromosome alignment, and kinetochore attachments (Balboula and Schindler, 2014) Ongoing error correction due to Aurora B/C activity in early meiosis, which is gradually shut off (Yoshida et al., 2015). Localization of BubR1 (Touati et al., 2015). Aurora C localized to inter- chromatid axis by H3 phosphorylation mark, role in chromosome condensation (Nguyen et al., 2014).

### Mps1: regulating kinetochore-microtubule attachments and the cohesin protector

Mps1, which can be viewed as the most upstream component of the SAC, also plays a central role in other aspects of mitosis and meiosis (Liu and Winey, 2012; Ciliberto and Hauf, 2017). *S. cerevisiae MPS1* was originally identified as being important for the duplication of the yeast centrosome/spindle pole body (Winey et al., 1991) but later found to also function in the SAC (Weiss and Winey, 1996). The *S. pombe* Mps1 homolog, Mph1, is also critical for the SAC, but unlike *S. cerevisiae* Mps1, Mph1 is not required for SPB duplication, and therefore *MPH1* is not an essential gene (He et al., 1998). This kinase plays a conserved and key role in the SAC, phosphorylating MELT repeats in the outer kinetochore scaffold protein Spc105/Spc7/Knl1, which serves as a platform for other SAC proteins to generate the MCC (see Musacchio, 2015; Sacristan and Kops, 2015 for reviews). In some organisms, Mps1 has been shown to be required together with Aurora B kinase for error correction of incorrect microtubule-kinetochore attachments and for the centromeric localization of Sgo1 (van der Waal et al., 2012; Williams et al., 2016), which protects centromeric cohesin from Wapl-dependent cohesin removal in mammalian mitotic prophase. However, Mps1 is not essential for localization of the other shugoshin paralog, Sgo2 in mammals, which does not contribute to mitotic cohesin protection (van der Waal et al., 2012).

#### Mps1 in yeast meiosis

As in mitosis, *S. cerevisiae* Mps1 is essential for spindle pole body duplication and chromosome segregation during meiosis (Straight et al., 2000). Hypomorphic *mps1* mutations that support vegetative growth have catastrophic effects on meiosis, indicating that meiosis is especially reliant on Mps1 (Straight et al., 2000; Meyer et al., 2013). Elegant live cell imaging in meiotic cells showed that one critical role of Mps1 in chromosome segregation during meiosis is to generate force-bearing attachments of kinetochores to microtubules (Meyer et al., 2013). The behavior of chromosomes in hypomorphic *mps1* mutants in meiosis indicates that Mps1 achieves this by converting the attachment of kinetochores to the sides (lateral) to the ends (end-on) of microtubules.

In addition, although anchor-away or kinase inhibition experiments revealed that *S. cerevisiae* Mps1 is dispensable for pericentromeric cohesin protection during meiosis I, conversely, during meiosis II it becomes important for this process, through maintaining the localization of shugoshin-PP2A, as described above (Argüello-Miranda et al., 2017). Indeed, APC/C^Cdc20^ –dependent degradation of both shugoshin-PP2A and Mps1 is required for chromosome segregation at meiosis II, indicating that inactivation of Mps1 contributes to the deprotection of pericentromeric cohesin during meiosis II (Jonak et al., 2017). These findings are suggestive of a hand-off between Bub1 and Mps1 in cohesin protection, with the former kinase localizing Sgo1 during meiosis I and the latter kinase localizing Sgo1 during meiosis II. Whether this is a strict division of labour, or if there is interplay between these kinases during meiosis, as is well established during mitosis (London et al., 2012; Shepperd et al., 2012; Yamagishi et al., 2012), is not currently clear. Similarly, the relevant target of Mps1 and the requirement of H2A-S121P during meiosis II remain unknown.

#### Mps1 in mouse oocytes

In mouse oocytes, Mps1 kinase activity is essential for proper chromosome segregation (Touati et al., 2015). Anaphase I onset takes place before chromosomes have had time to congress to the metaphase plate, and gross missegregations lead to the generation of aneuploid oocytes that cannot give rise to viable embryos after fertilization. Without fully functional Mps1, the SAC is abolished: Mad2 is not recruited to unattached kinetochores, and the APC/C is activated precociously. Interestingly, even though anaphase I onset is strongly accelerated, oocytes still establish stable end-on kinetochore-microtubule attachments in the absence of functional Mps1, indicating that Mps1 is not required for initial attachments (Hached et al., 2011; Touati et al., 2015). Preventing anaphase I onset allows oocytes to congress chromosomes to the metaphase plate, except those that are close to the poles, which seem to remain stably attached to one pole without being able to establish end-on attachments coming from the other pole (Hached et al., 2011). Even when accelerated anaphase I onset is prohibited, oocytes cannot establish correct attachments of bivalents at the poles, indicating that error correction is not properly functional in oocytes without Mps1. Indeed, Aurora B kinase, which is required for severing wrongly attached microtubules, is mis-localized in oocytes not harboring fully functional Mps1, and Mps1 localization to the kinetochore is decreased without Aurora B kinase activity (Hached et al., 2011; El Yakoubi et al., 2017).

Apart from Mps1's well established role in checkpoint control, Mps1 kinase activity in oocytes is essential for correct Sgo2 localization. In the absence of Mps1 kinase activity, Sgo2 recruitment to the centromere is reduced in meiosis I. This leads to failures in proper centromeric cohesin protection and the separation of some sister chromatids at the first meiotic division. Importantly, this recruitment of Sgo2 occurs independently of the canonical Histone H2A-T120 mark, and does not require kinetochore localization of Mps1 (Figure 3). Hence, oocytes devoid of correctly localized Mps1 but still harboring Mps1 kinase activity, are defective in checkpoint control, but not cohesin protection (El Yakoubi et al., 2017). The molecular targets of Mps1 kinase for Sgo2 localization at the centromere and for cohesin protection in meiosis I are still unknown.

#### Summary

Mps1 is required for spindle pole duplication in yeast meiosis, but not for centrosome duplication in mammalian oocytes, which divide without centrosomes. Cohesin protection in the first meiotic division in mouse oocytes depends on Mps1 kinase-dependent localization of Sgo2 to the centromere, whereas in *S. cerevisiae*, Mps1 kinase is dispensable for cohesin protection, but required for sister chromatid segregation in meiosis II. Mps1's role for meiosis II in oocytes is still unknown.

### Mad3/BubR1: not requiring any kinase activity

Mad3 in yeast and its mammalian counterpart, BubR1, are both components of the MCC, with homology to Bub1 (see (Musacchio, 2015; Sacristan and Kops, 2015) for reviews). Unlike mammalian BubR1, yeast Mad3 lacks a kinase domain. Mammalian BubR1 is an untypical pseudo-kinase, which is thought to have maintained conserved residues for a kinase domain throughout evolution for structural reasons (Suijkerbuijk et al., 2012). Catalytic activity of BubR1 is not required for any of its functions, at least in mammalian cells. BubR1 is a component of the MCC and therefore essential for the spindle checkpoint. It binds to unattached kinetochores through interaction with Bub3, which is recruited to phosphorylated MELT repeats of Knl1 by the Bub3-Bub1 complex. The MCC is then thought to be formed at the unattached kinetochore with simultaneously recruited Cdc20 and Mad2.

#### Mad3 in yeasts

Unlike Mad2 or Mad1, Mad3 does not appear to be essential for meiosis in *S. cerevisiae* as cells lacking *MAD3* exhibit normal spore viability (Shonn et al., 2000, 2003). Cells lacking *MAD3* fail to activate the SAC in response to kinetochore-microtubule attachment defects in the same way as cells lacking *MAD2*, however, Mad3 does not seem to share the additional roles of Mad2 in orienting homologs during meiosis I (Shonn et al., 2003). Interestingly, Mad3 becomes critical in cells carrying a single chromosome that does not form crossovers and therefore does not generate tension during meiosis I. This non-exchange chromosome relies on a Mad3-mediated prophase delay that occurs during every meiosis for their proper segregation (Cheslock et al., 2005). Therefore current evidence supports the idea that the primary function of Mad3 in *S. cerevisiae* meiosis is to prolong prophase, as part of the SAC. Interestingly, human BubR1 was able to compensate for this function of Mad3 in *S. cerevisiae* meiosis (Cheslock et al., 2005).

#### BubR1 in mouse oocytes

Given the importance for BubR1 in mitotic SAC control it is not surprising that it is equally essential for meiotic SAC control. As for other SAC components, its loss of function leads to accelerated meiosis I in oocytes, with such high mis-segregation events that mice with a conditional, oocyte -specific knock-out of BubR1 are sterile. Importantly though, BubR1 has a role in promoting progression through anaphase I, independently of its SAC-related role. Oocytes devoid of BubR1 undergo accelerated anaphase I onset, degrade cyclin B1 and securin, and are then delayed in chromosome segregation and polar body extrusion (Touati et al., 2015). It is unknown whether this additional role of BubR1 is restricted to oocyte meiosis, or is equally promoting anaphase onset in mitosis. As BubR1's role for promoting progression through anaphase I is not essential and oocytes eventually manage to exit meiosis I, this function of BubR1, if conserved, may have been missed in somatic cells, due to the much more rapid progression through mitosis, compared to oocyte meiosis.

In oocytes, BubR1 was proposed to be required for the extended prophase arrest before entry into the first meiotic division (Homer et al., 2009), but this was not confirmed with a knock-out approach and is therefore most probably due to the morpholino-knock-down technique that has been used, without simultaneous assessment of knock-down levels in individual cells. Oocytes completely devoid of BubR1 remain correctly arrested in prophase I and upon release, enter meiosis I on time, indicating that BubR1 is not required for prophase I arrest (Touati et al., 2015).

The mitotic role of BubR1 in stabilizing kinetochore-microtubule attachments is conserved in oocytes. But contrary to mitosis, BubR1 does not need to be localized to kinetochores to stabilize microtubules in oocytes (Touati et al., 2015). In mitosis, BubR1 recruits PP2A-B56 phosphatase to kinetochores, where it counteracts the microtubule-destabilizing activity of Aurora B (Suijkerbuijk et al., 2012; Kruse et al., 2013; Xu et al., 2013). In oocytes devoid of BubR1, stabilization of kinetochore fibers cannot be rescued by inhibiting Aurora B kinase activity, indicating that BubR1 promotes stabilization of kinetochore fibers in oocytes in a distinct manner, in the cytosol (Touati et al., 2015).

#### Summary

Both yeast Mad3 and mammalian BubR1 are components of the MCC. In *S. cerevisiae* meiosis, Mad3 is required for the segregation of non-exchange chromosomes by delaying prophase I. Cytosolic BubR1 in oocytes is necessary to stabilize kinetochore-microtubule interactions. BubR1 furthermore promotes progression through anaphase I.

### Ipl1/Ark1/Aurora B is important for multiple steps in meiosis

In mitosis, the role of the Aurora B kinase in the SAC signaling cascade is still controversial and beyond the scope of this review. As mentioned above, Aurora B is at least in part required for full Mps1 activity and thereby for prolonged maintenance of SAC arrest. Being an essential component of the chromosomal passenger complex (CPC), Aurora B destabilizes erroneous kinetochore-microtubule attachments that are not under tension from the bipolar spindle, to permit establishment of correct attachments, and controls the conversion of lateral to end-on attachments in mitosis (Kalantzaki et al., 2015; Shrestha et al., 2017), for review see (van der Horst and Lens, 2014; Krenn and Musacchio, 2015). Independent of its potential role in SAC signaling, Aurora B maintains SAC activity by creating unattached kinetochores. Besides error correction, Aurora B is important for chromosome condensation and cytokinesis in mitosis (for review see van der Horst and Lens, 2014).

#### Ipl1/Ark1 in yeast

Budding yeast Aurora B kinase has been implicated in many processes that are essential for successful meiosis. Meiosis-specific depletion of budding yeast Aurora B kinase, Ipl1, revealed that it is required for the biorientation of both homologs in meiosis I and sister chromatids during meiosis II, suggesting that it is important for error correction in both meiotic divisions in addition to mitosis (Monje-Casas et al., 2007). This was later confirmed through live cell imaging studies (Meyer et al., 2013). Ipl1 is also important earlier, during meiotic prophase, where it is important for coordination of meiotic processes to prevent premature chromosome segregation (Kim et al., 2013). Several studies observed an uncoupling of meiotic events in Ipl1-depleted cells (Jordan et al., 2009; Shirk et al., 2011; Kim et al., 2013; Newnham et al., 2013). Ipl1 was originally thought to be important for timely disassembly of the normally prophase-specific synaptonemal complex (SC) since SCs were found to co-exist with the meiosis I spindle, which should form only upon prophase exit, in Ipl1-depleted cells (Jordan et al., 2009). However, later studies showed that this phenotype was largely due to assembly of the meiosis I spindle already in prophase (Shirk et al., 2011; Kim et al., 2013; Newnham et al., 2013). How Ipl1 prevents spindle assembly in prophase remains unclear. Ipl1 localizes on microtubules next to the yeast centrosome (called the spindle pole body) and dissociation of Ipl1 at prophase I exit occurs coincident with spindle assembly (Kim et al., 2013). Therefore, one possibility is that Ipl1 acts to destabilize microtubules near SPBs in a similar manner to disruption of kinetochore microtubules during error correction (Kim et al., 2013). A further function of Ipl1, which also acts to prevent premature chromosome segregation, is to trigger the shedding of the outer kinetochore during meiotic prophase, thereby abolishing the ability of kinetochores to interact with microtubules and promoting assembly of the monopolin complex, which directs kinetochore co-orientation during meiosis I (Kim et al., 2013; Meyer et al., 2015). In addition to its microtubule/kinetochore-related functions, Ipl1 is important for the maintenance of centromeric cohesion, potentially due to a failure to maintain the protective Sgo1-PP2A complex at centromeres (Monje-Casas et al., 2007; Yu and Koshland, 2007).

*S. pombe* Aurora B kinase, Ark1, also plays multiple essential roles that impact meiotic chromosome segregation. In addition to promoting homolog biorientation, Ark1 is essential for sister kinetochore co-orientation in this organism (Hauf et al., 2007). Interestingly, loading of Ark1 to kinetochores relies on *S. pombe* shugoshin 2 (Sgo2), which is distinct from shugoshin 1 (Sgo1) that protects cohesion in this organism (Kawashima et al., 2007). Ark1 suppresses the attachment of microtubules from opposite poles to binding sites on the same kinetochore (merotelic attachment) during meiosis I, in a manner dependent on the presence of chiasmata (Sakuno et al., 2011). During this chiasma-dependent realignment process, Ark1 relocates to the inner centromere on the inside face of the bivalent, away from the site of microtubule attachment (Sakuno et al., 2011). This suggests that Ark1 is responsive to tension across homologs, but the molecular mechanism of how this tension is sensed remains completely unknown.

#### Aurora B in mouse oocytes

Mammalian oocytes harbor Aurora B and the closely related Aurora C kinase (Sasai et al., 2004), which is lacking certain destruction motifs found in Aurora B, and is therefore more stably expressed (Schindler et al., 2012). Oocyte-specific Aurora C knock-out only leads to subfertility, showing that Aurora C is important for meiotic maturation, but not essential to generate oocytes that can be fertilized (Schindler et al., 2012), suggesting a certain redundancy in the roles of these two kinases. It is mainly Aurora C that promotes localized CPC activity, chromosome alignment and establishment of kinetochore attachments, required for efficient meiotic progression (Balboula and Schindler, 2014), probably due to higher protein stability compared to Aurora B.

Studies on error correction in meiosis have used small molecule inhibitors that do not allow to distinguish between the respective roles of Aurora B and C, but Aurora B/C kinase-dependent error correction was shown to take place in prometaphase I, when attachments undergo multiple attachment cycles (Kitajima et al., 2011; Yoshida et al., 2015). Aurora B/C activity continuously decreases as oocytes progress into metaphase I. Artificially down-regulating Aurora B/C during meiotic maturation was shown to help oocytes establish end-on attachments and prevent aneuploidies (Yoshida et al., 2015). Nevertheless, this does not mean that Aurora B/C's role in meiosis is expendable or even harmful for establishing correct attachments in general, as attested by expressing a dominant negative version of Aurora C in oocytes (Balboula and Schindler, 2014). Also, the importance of Aurora B/C to detect attachments that are not under tension before metaphase-to-anaphase transition has not yet been addressed in oocytes.

Maybe in agreement with a higher stability of Aurora C, the kinase is not only detected in the centromere region but also at the axis in between the sister chromatids, in meiosis I. Aurora C as part of the CPC is recruited by the Haspin-dependent phospho-Histone H3 mark to the centromere region and interchromatid axis in meiosis I (Nguyen et al., 2014). Aurora C's role at the interchromatid axis is still unknown, but may be related to its role in chromosome condensation (Nguyen et al., 2014).

#### Summary

Ipl1, Ark1, and Aurora B/C fulfill multiple roles in meiosis, such as for chromosome condensation, preventing premature chromosome segregation, and cytokinesis. The kinase counteracts the establishment of stable, end-on attachments of kinetochores to microtubules, probably in all organisms as part of error correction of tension-less attachments.

### The role of SAC kinases in CSF arrest

It was proposed that in addition to Mad1 and Mad2 (Tunquist et al., 2003), the SAC kinase Bub1 participates in establishing CSF arrest by down-regulating Cyclin E-Cdk2, in *Xenopus laevis* oocytes, with the caveat that these experiments were performed using overexpressed proteins (Tunquist et al., 2002). A role in CSF arrest for endogenous SAC kinases has not been shown. There is enough evidence in mammalian oocytes excluding an essential role for SAC kinases in CSF arrest, both using conditional knock-out mouse models and expression of dominant negative constructs (Tsurumi et al., 2004; Touati et al., 2015). This indicates that beyond potential other functions in meiosis II, such as SAC control, Sgo2 localization, and stabilization of kinetochore-microtubule interactions, SAC kinases are not implicated in establishing or maintaining a CSF arrest. Future work will show whether SAC kinases have additional, as yet unknown, roles in meiosis II.

## Conclusion

The specificities of the meiotic cell division require adaptation of known regulatory mechanisms that govern somatic cell divisions. Kinases that are important for mitotic SAC control fulfill important additional roles in meiosis, independent of a functional SAC (see Table 1 for a summary of the different roles SAC kinases play in meiosis). Well-known model organisms such as mouse, yeast and *Drosophila*, emerging model systems, and comparative evolutionary studies will help us to obtain a better picture of the multiple steps regulated by these kinases. Importantly, even though the result of meiosis is the same in all models (the generation of haploid cells), details in the molecular pathways ensuring the correct segregation of chromosomes and sister chromatids may vary. These differences provide key insight that will help us understand the essential parts and targets of each pathway for the generation of euploid gametes.

## Author contributions

KW has written most of the part of this review dealing with meiosis in higher organisms, and AM has written most of the part on meiosis in yeast. Both authors have corrected the whole manuscript.

### Conflict of interest statement

The authors declare that the research was conducted in the absence of any commercial or financial relationships that could be construed as a potential conflict of interest. The reviewer DF declared a shared affiliation, with no collaboration, with one of the authors, KW, to the handling Editor.

## Abbreviations

SAC: Spindle Assembly Checkpoint
Bub1, 3: Budding uninhibited by Benomyl 1, 3
BubR1: Bub1 related
Mad1-3: Mitotic arrest deficient 1-3
Mps1: Monopolar Spindles 1
Ipl1: Increase-in-ploidy 1
Ark1: Aurora-related kinase 1
SPB: Spindle Pole Body
APC/C: Anaphase Promoting Complex/ Cyclosome
MPF: M-phase Promoting Factor
CSF: Cytostatic Factor
GVBD: Germinal Vesicle Breakdown
PKA: Protein Kinase A
Cdk1, 2: Cell division kinase 1, 2
Cdc20: Cell division cycle 20
Zp3: Zona pellucida 3
Knl1: Kinetochore null 1
MCC: Mitotic Checkpoint Complex
Sgo1, 2: Shugoshin 1,2
PP2A: Protein phosphatase 2A
H2A: Histone H2A
Mph1: Mps1p-like pombe homolog
Wapl: Wings apart-like protein homolog.
